# Supplementary material for: NCP activates chloroplast transcription by controlling phytochrome-dependent dual nuclear and plastidial switches
Source: Nat Commun. 2019 Jun 14;10:2630. doi: 10.1038/s41467-019-10517-1 (PMC6570768; doi:10.1038/s41467-019-10517-1)
Supplement: Supplementary file 1 — Supplementary Information [file 41467_2019_10517_MOESM1_ESM.pdf]

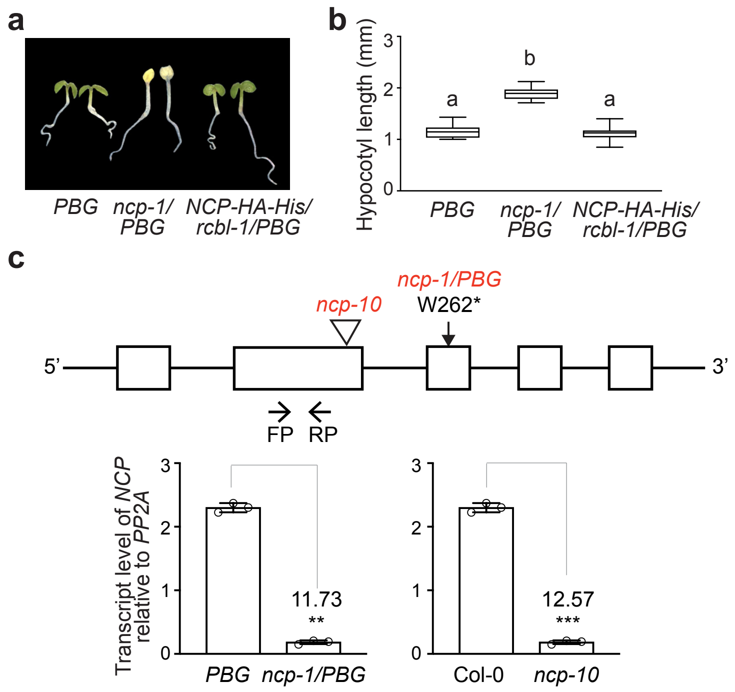

Supplementary Fig. 1. Expressing HA and His tagged *At*2g31840, *NCP-HA-His*, rescues *ncp-1/PBG*. a, Representative images of 4-day-old *PBG*, *ncp-1/PBG*, and *NCP-HA-His/ncp-1/PBG* seedlings grown in 10 μmol m^{-2} s^{-1} R light. **b**, Box-and-whisker plot showing hypocotyl length measurements for seedlings from **a**. The boxes represent from the 25th to the 75th percentiles, the bars equal the median values. Samples with different letters have statistically significant differences in hypocotyl length (ANOVA, Tukey's HSD, $p \leq 0.0001$, $n > 20$). **c**, qRT-PCR analyses of the steady-state mRNA levels of *NCP* in 4-d-old *PBG*, *ncp-1/PBG*, Col-0, and *ncp-10* seedlings grown under 10 μmol m^{-2} s^{-1} R light. Schematic of the intron-exon structure of NCP with the position of mutations are shown. The position for qPCR primers (FP and RP) is located with arrows.The transcript levels of *NCP* were calculated relative to those of *PP2A*. Error bars represent SD of three biological replicates. Asterisks indicate a statistically significant difference in the transcript levels of the bridged columns (Student's *t*-test, **, $p \leq 0.01$, *** $p \leq 0.001$). The source data underlying the hypocotyl measurements in **b** and the qRT-PCR data in **c** are provided in the Source Data file.

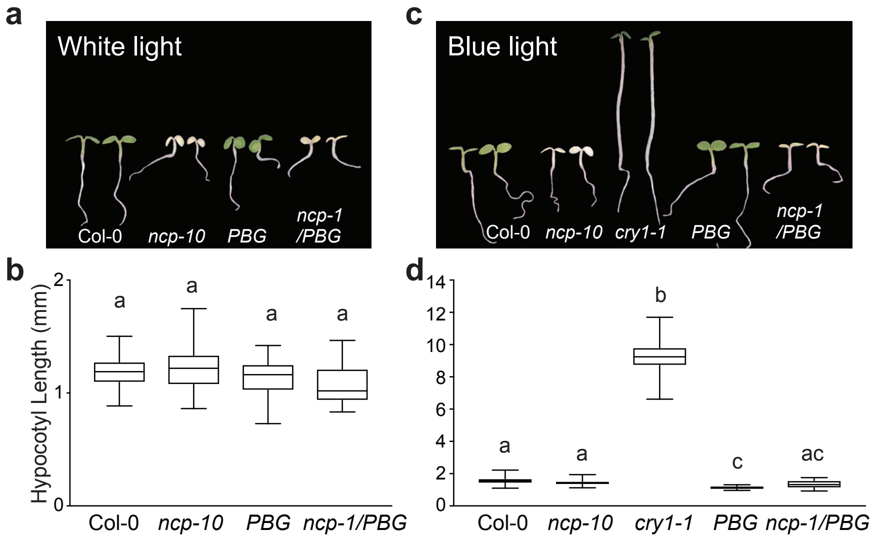

Supplementary Fig. 2. *ncp* mutants exhibit normal hypocotyl responses to blue and white light.

a, Representative images of 4-d-old Col-0, *ncp-10*, *PBG*, and *ncp-1/PBG* seedlings grown in 33 μmol m^{-2} s^{-1} white light. **b**, Box-and-whisker plots for hypocotyl length measurements of seedlings in **a**. **c**, Representative images of 4-d-old Col-0, *ncp-10*, *cry1-1*, *PBG*, and *ncp-1/PBG* seedlings grown in 10 μmol m^{-2} s^{-1} blue light. **d**, Box-and-whisker plots for hypocotyl length measurements of seedlings in **c**. For **b** and **d**, the boxes represent from the 25th to the 75th percentiles, and the bars equal the median values. Samples with different letters indicate statistically significant differences in hypocotyl length (ANOVA, Tukey's HSD, $p \leq 0.001$, $n > 40$). The source data underlying the hypocotyl measurements in **b** and **d** are provided in the Source Data file.

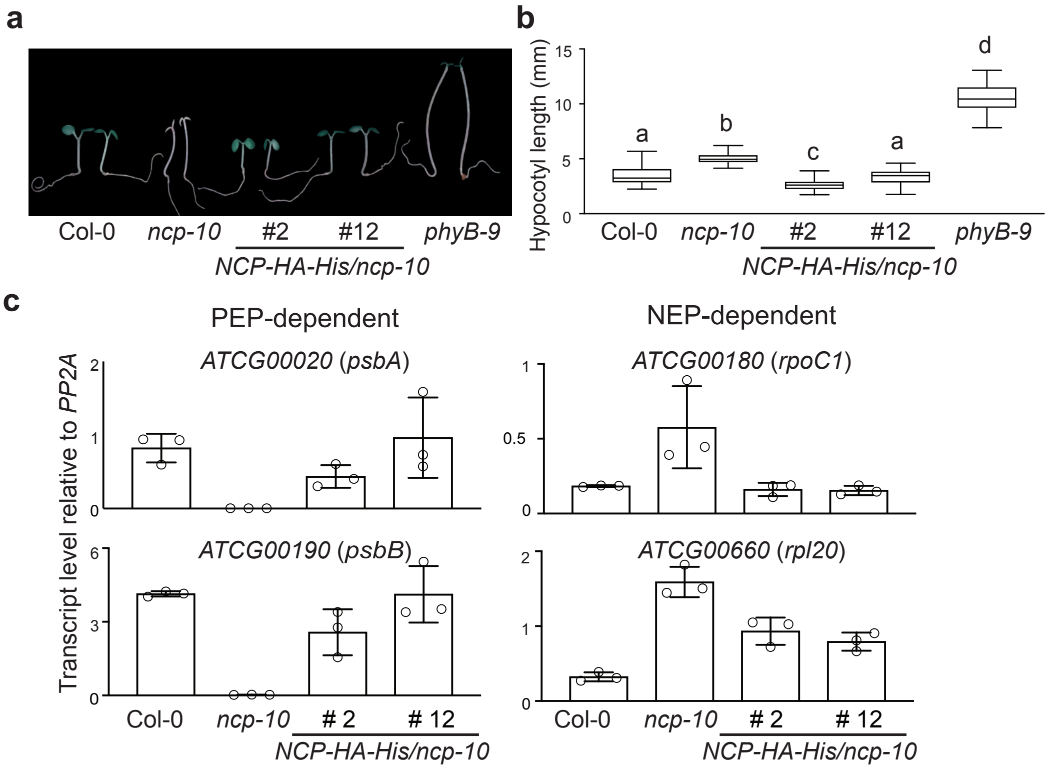

Supplementary Fig. 3. Expressing HA and His tagged *NCP-HA-His* rescues *ncp-10.*
a, Representative images of 4-d-old Col-0, *ncp-10*, *NCP-HA-His/ncp-10* line *#2* and *#12*, and *phyB-9* seedlings grown in 10 μmol m^{-2} s^{-1} R light. **b**, Box-and-whisker plots showing hypocotyl length measurements of seedlings from **a**. The boxes represent from the 25th to the 75th percentiles, and the bars equal the median values. Samples with different letters exhibit statistically significant difference in hypocotyl length (ANOVA, Tukey's HSD, $p \leq 0.001$, $n > 30$). **c**, qRT-PCR analyses of the steady-state mRNA levels of selected PEP-dependent and NEP-dependent genes in 4-d-old Col-0, *ncp-10*, *ncp-10/NCP-HA-His #2,* and *ncp-10/NCP-HA-His #12* grown in 10 μmol m^{-2} s^{-1} R light.Transcript levels were calculated relative to those of *PP2A*. Error bars represent SD of three biological replicates. The source data underlying the hypocotyl measurements in **b** and the qRT-PCR data in **c** are provided in the Source Data file.

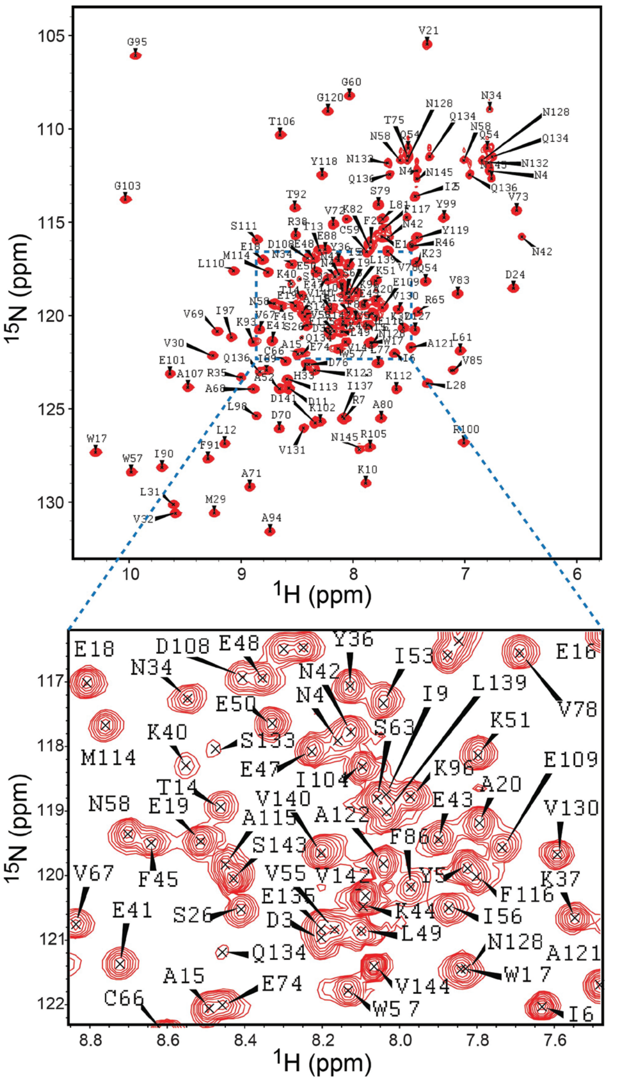


```
     NMR   3        10         20         30         40         50
SEQUENCE   HMDNYIRPIK DLTTAEWEEA VFKDISPLMV LVHNRYKRPK ENEKFREELE
     NCP   208      215        225        235        245        255

     NMR            60         70         80         90         100
SEQUENCE   KAIQVIWNCG LPSPRCVAVD AVVETDLVSA LKVSVFPEII FTKAGKILYR
     NCP            265        275        285        295        305

     NMR            110        120        130        140  145
SEQUENCE   EKGIRTADEL SKIMAFFYYG AAKPPCLNGV VNSQEQIPLV DVSVN
     NCP            315        325        335        345  350
```

Supplementary Fig. 4. ^{1}H-^{15}N HSQC spectrum of the NCP Trx-like domain.
The sequence of the NMR construct is shown below the NMR spectrum. Resonance assignments of individual signals reflect the residue numbers within the NMR construct. The residue numbers within the full-length NCP protein are labeled in blue beneath the sequence.

a

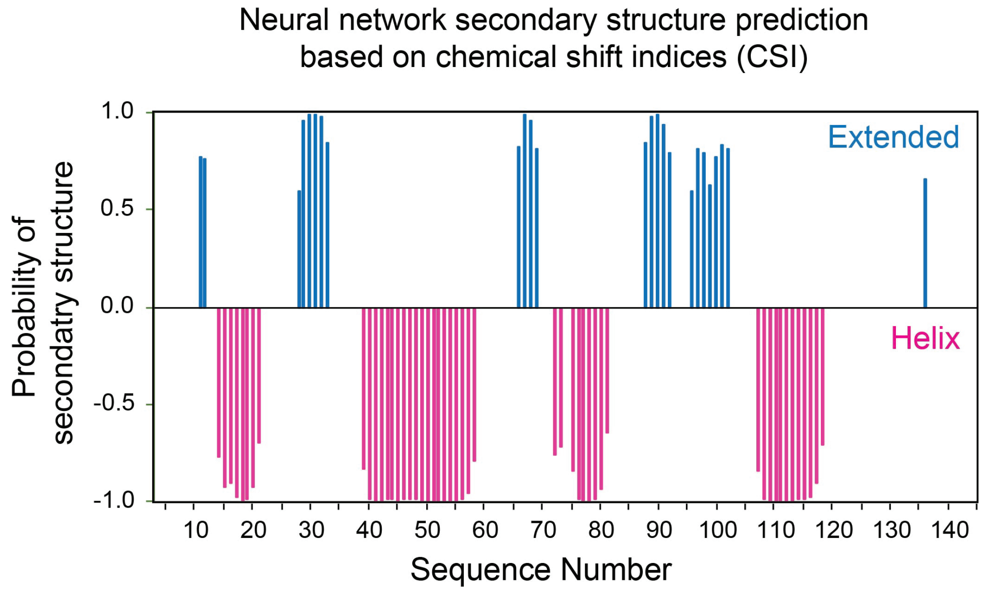

b

```
NCP            208     215          225          235          245          255
NMR              3      10           20           30           40           50
SEQUENCE       DNYIRPIK DLTTAEWEEA VFKDISPLMV LVHNRYKRPK ENEKFREELE
PREDICTED_SS   LLLLLLLL EELHHHHHHH HLLLLLLEEE EEELLLLLHH HHHHHHHHHH
CONFIDENCE     08899984 5545889998 3478997199 9966899869 9999999999

NCP                       265          275          285          295          305
NMR                        60           70           80           90          100
SEQUENCE       KAIQVIWNCG LPSPRCVAVD AVVETDLVSA LKVSVFPEII FTKAGKILYR
PREDICTED_SS   HHHHHHHHLL LLLLLEEEEL LHHLHHHHHH HLLLLLLEEE EELLLEEEEE
CONFIDENCE     9999999548 9998669965 6543699998 2689551699 8507716525

NCP                       315          325          335          345    350
NMR                       110          120          130          140    145
SEQUENCE       EKGIRTADEL SKIMAFFYYG AAKPPCLNGV VNSQEQIPLV DVSVN
PREDICTED_SS   EELLLLHHHH HHHHHHHHLL LLLLLLLLLL LLLLLELLLL LLLLL
CONFIDENCE     6636687999 9999997468 9999879971 6898236778 88880
```

Supplementary Fig. 5. Secondary structure analysis of the NCP Trx-like domain.
a, Neural network secondary structure prediction based on the chemical shift indices (CSI) analysis of the NCP Trx-like domain. The probability of each residue adopting an extended or helical conformation is plotted in blue or pink, respectively. **b,** The predicted secondary structures and confidence levels along the primary protein sequence. Residue numbers in the NMR construct and full-length NCP are colored in black and blue respectively. Residues predicted in the extended conformation are colored in blue, while those in the helical conformation are colored in pink.

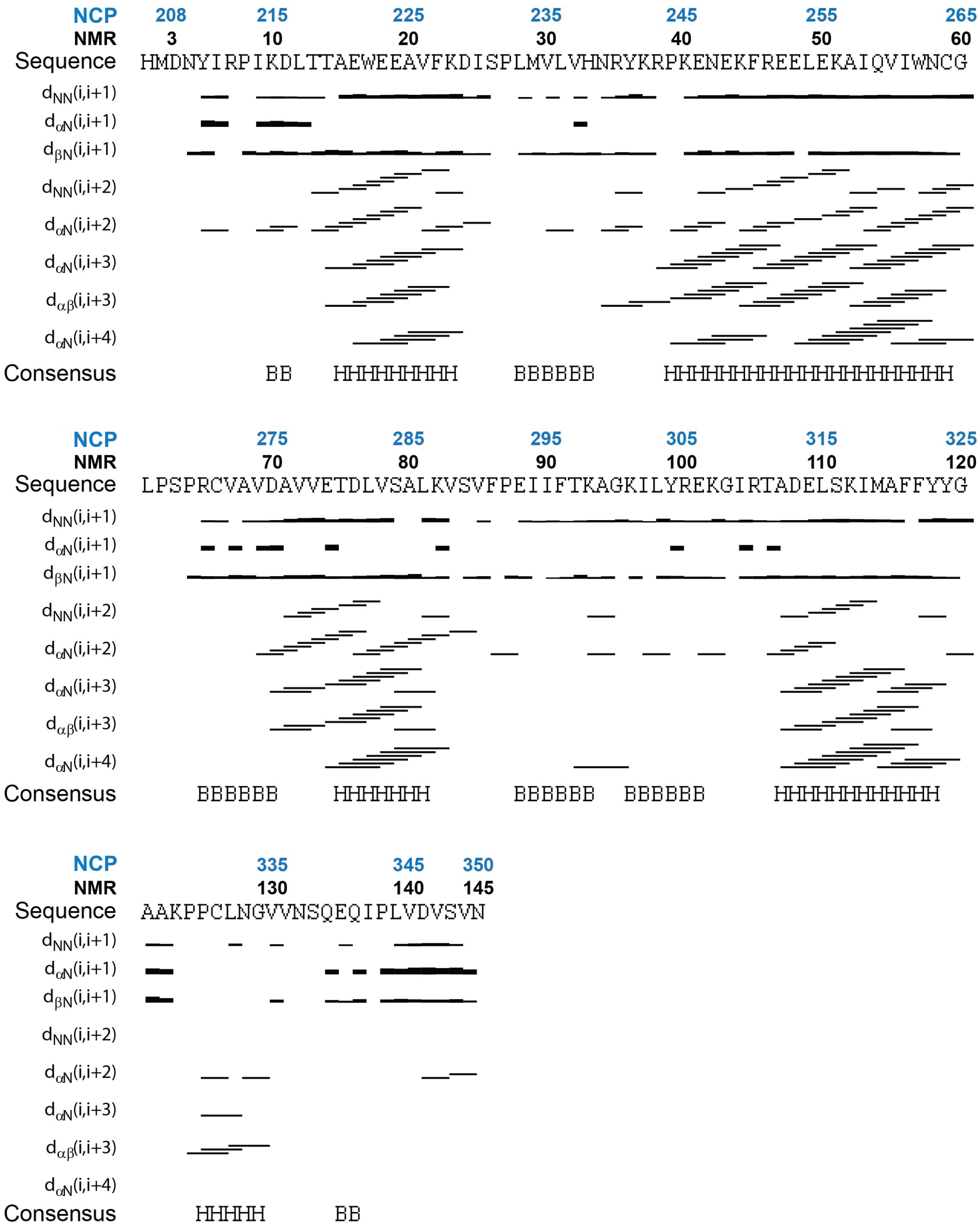

Supplementary Fig. 6. Secondary structure determination of the NCP Trx-like domain based on the NOE pattern.

The presence of β-strands (extended conformations) is reflected by very strong NOE signals of d_{aN}(i, i+1) (indicated by thick bars), whereas the presence of helices is reflected by the characteristic NOEs of $d_{\alpha\beta}$(i, i+3), $d_{\alpha N}$(i, i+3) and $d_{\alpha N}$(i, i+4).

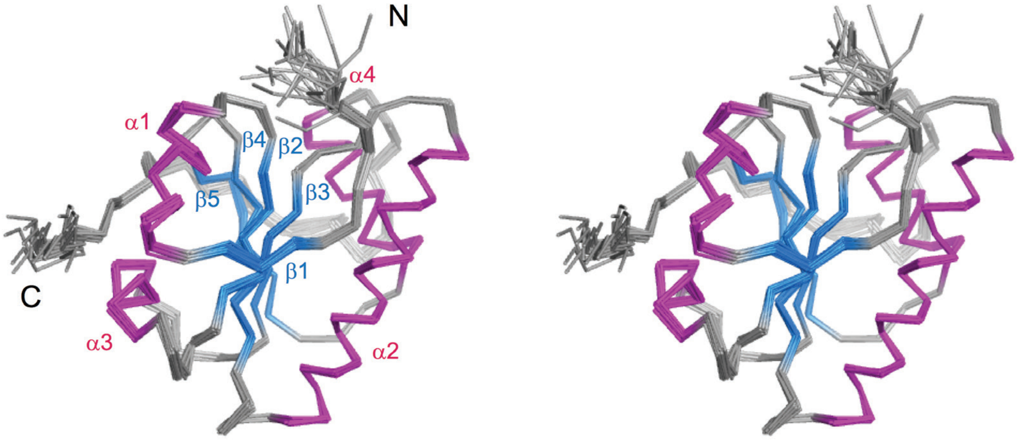

Supplementary Fig. 7. A stereo view of the NMR structural ensemble of the NCP Trx-like structures. Alpha-helices and beta-strands are colored in magenta and blue, respectively.

Supplementary Table 1. Primers used for making plasmid constructs

Accession	Gene name	Vector name	Forward primer	Reverse primer
*At*2g31840	*NCP-HA-His*	pCHF1	TTCTTCTATAAAACAATACCATGATTCTTCCATTTTCGACACAGTTCACTTGC	GGGGGTGGGGGTGGGGGTGGGGGTGGGATTCACACTTACATCGACTAAAGG
*At*2g31840	*NCP-CGP-Flag*	pCHF3	ACGGGGGACGAGCTCGGTACCCGGGATGATTCTTCCATTTTCGAC	GTGTTGGAGTAGGCTCTAGAATATTCACACTTACATCGACTA
At2g31840	*NCP-FL*	pCMX-PL2	TCGAGAAGCTTGATATCGAATTCATGATTCTTCCATTTTCGAC	TACTAGCTAGCTGGCCAGGATCCTCAGTGATGGTGATGGTG
At2g31840	*NCPΔ48*	pCMX-PL2	TCGAGAAGCTTGATATCGAATTCATGTCCTCCTCGAAGTGG	TACTAGCTAGCTGGCCAGGATCCTCAGTGATGGTGATGGTG
*At*2g31840	*NCP-FL*	pET15b	ATACATATGATGATTCTTCCATTTTC	TTGCTCGAGTTAATTCACACTTACATCGAC
*At*2g31840	*NCPΔ206*	pET15b	TAACATATGGGAGATGATAGTGAGAAGGA	TTGCTCGAGTTAATTCACACTTACATCGAC
At4g28590	*RCBΔ98*	pET28a	CAGCAAATGGGTCGCGCTAGTGAAGCAGACGCTGTG	CTCGAGTGCGGCCGCACTAACAGTACGGGGTTAC
At4g28590	*RCBΔ197*	pET28a	CAGCAAATGGGTCGCGCTGAGATTGATGATAGTTGGG	CTCGAGTGCGGCCGCACTAACAGTACGGGGTTAC

Supplementary Table 2. qRT-PCR primers for the plastidial-encoded genes examined in this study

Accession	Gene name	Primer for cDNA synthesis	Primer pair for qRT-PCR
ATCG00020	*psbA*	TAGATGGAGCCTCAACAGCAGCTA	ACATTTCTTCTTAGCGGCTT<br>CGTCCTTGACTATCAACTACTGA
ATCG00680	*psbB*	CATCCAAATCTGGATCAATACCAG	ACATTTCTTCTTAGCGGCTT<br>CGTCCTTGACTATCAACTACTGA
ATCG00490	*rbcL*	CTTCACAAGCAGCAGCTAGTTCAGG	GGAGATGATTCTGTACTACAAT<br>GTCCCTCATTACGAGCTTGTAC
ATCG00190	*rpoB*	CAATGATAGTGGTACCAAGTACTTC	CTAGTGGACATTATGCACTTGT<br>CAGATTTATAAGTAAGCATCTCTTG
ATCG00180	*rpoC1*	GTATAGCTTCCTCGATTTCTCG	GATGCAATTGGAGCTTATCG<br>CGATAGGAACTTCTCTTGAAGC
ATCG00660	*rpl20*	TGTGCAAGTATTTTCCGATTAAG	GAGCTTTAGTTTCGGCTCATC<br>AATTACGGCATTTATTCGAGTG

Supplementary Table 3. qRT-PCR primers for the nuclear genes examined in this study.

Accession	Gene name	Forward primer	Reverse primer
AT1G69960	*PP2A*	TATCGGATGACGATTCTTCGTGCAG	GCTTGGTCGACTATCGAATGAGAG
AT2G31840	*NCP*	GAAGCGAGTCTCGATGATCC	GCTTCTGCATCTTCCTCCTC

Supplementary Table 4. Gene ID or transcript ID for phylogenetic analysis of NCP and RCB homologues.

Taxas	Gene name	Gene ID	Transcript Identifier	PAC ID	Source	1KP Identifier
**Angiosperm**						
Amborella trichopoda	*NCP*	ATR_00066G00940	evm_27.model.AmTr_v1.0_scaffold00066.94 CDS	-	Genome, Amborella Genome Project	-
	RCB	ATR_00066G01920	evm_27.model.AmTr_v1.0_scaffold00066.192 CDS	-	Genome, Amborella Genome Project	-
Arabidopsis thaliana	*NCP*	AT2G31840.1	-	19643553	Genome, Phytozome	-
	RCB	AT4G28590.1	-	19645001	Genome, Phytozome	-
Oriza sativa	*NCP*	LOC_Os05g34470	-	24152372	Genome, Phytozome	-
	RCB	LOC_Os01g61320	-	24114913	Genome, Phytozome	-
**Gymnosperm**						
Ginkgo biloba	*NCP*	-	SGTW_scaffold_2001407 Ginkgo_biloba	-	Transcriptome, 1KP	SGTW
	RCB	-	SGTW_scaffold_2037234 Ginkgo_biloba	-	Transcriptome, 1KP	SGTW
Picea abies	*NCP*	MA_875929	-	-	Congenie, Genome	-
	RCB	MA_85974	-	-	Congenie, Genome	-
Gnetum montanum	*NCP*	GMO00014786	GTHK-0020791-2011195-Gnetum_montanum_987	-	Transcriptome, 1KP	GTHK
	RCB	GMO00015329	GTHK-2055953-0021387-Gnetum_montanum_1122	-	Transcriptome, 1KP	GTHK
**Fern**						
Equisetum diffusum	*NCP/RCB*	-	CAPN-1001418-2006808-Equisetum_diffusum_996	-	Transcriptome, 1KP	CAPN

Psilotum nudum	*NCP/RCB*	-	QVMR-2010863-0072637-Psilotum_nudum_1272	-	Transcriptome, 1KP	QVMR
Angiopteris evecta	*NCP/RCB*	-	NHCM-0020880-2008213-Angiopteris_evecta	-	Transcriptome, 1KP	NHCM
**Lycophytes**						
Selaginella moellendorffii	*NCP/RCB*	SM00002G04620	404435	-	Genome, Phytozome	-
Pseudolycopodiella caroliniana	*NCP/RCB*	-	UPMJ-2011768-0026604-Pseudolycopodiella_caroliniana_1203	-	Transcriptome, 1KP	UPMJ
**Hornwort**						
Nothoceros vincentianus	*NCP/RCB*	-	TCBC-2019434-0029159-Nothoceros_vincentianus_1187	-	Transcriptome, 1KP	TCBC
**Liverwort**						
Sphaerocarpos texanus	*NCP/RCB*	-	HERT-0056768-2043911-Sphaerocarpos_texanus_930	-	Transcriptome, 1KP	HERT
**Moss**						
Physcomitrella patens	*NCP/RCB*	Pp3c3_26500	PHYPADRAFT_172419	-	Genome, Phytozome	-

"-" indicates not applicable.
